# A mouse model for SARS-CoV-2-induced acute respiratory distress syndrome

**DOI:** 10.1038/s41392-020-00451-w

**Published:** 2021-01-01

**Authors:** Weiqi Hong, Jingyun Yang, Zhenfei Bi, Cai He, Hong Lei, Wenhai Yu, Yun Yang, Changfa Fan, Shuaiyao Lu, Xiaozhong Peng, Xiawei Wei

**Affiliations:** 1 Laboratory of Aging Research and Cancer Drug Target, State Key Laboratory of Biotherapy and Cancer Center, National Clinical Research Center for Geriatrics, West China Hospital, Sichuan University, No. 17, Block 3, Southern Renmin Road, 610041 Chengdu, Sichuan People’s Republic of China; 2 National Kunming High-level Biosafety Primate Research Center, Institute of Medical Biology, Chinese Academy of Medical Sciences and Peking Union Medical College, Yunnan, China; 3 Division of Animal Model Research, Institute for Laboratory Animal Resources, National Institutes for Food and Drug Control, 102629 Beijing, China; 4 State Key Laboratory of Medical Molecular Biology, Department of Molecular Biology and Biochemistry, Institute of Basic Medical Sciences, Medical Primate Research Center, Neuroscience Center, Chinese Academy of Medical Sciences, School of Basic Medicine Peking Union Medical College, Beijing, China

**Keywords:** Infection, Cancer models

**Dear Editor,**

The COVID-19 pandemic has covered more than 200 countries and regions around the world since its outbreak in January 2020. To date, the SARS-CoV-2 virus has caused >1.2 million deaths. The mortality rate of COVID-19 is closely concerned with the clinical symptoms of the patients from mild-to-severe disease. Notably, in its most severe form, COVID-19 leads to life-threatening pneumonia and acute respiratory distress syndrome (ARDS), which is mostly accompanied by a hyperactive immune response called “cytokine storm” and has high death rates from 40 to 50%.^1^ To elucidate the mechanisms of COVID-19 immunity and pathogenesis, researchers have developed several SARS-CoV-2 mouse models using transgenic mouse lines expressing hACE2 by the nasal inoculation of SARS-CoV-2.^2,3^ However, these models showed mild-to-moderate interstitial pneumonia with mononuclear cell infiltration,^2,3^ the pathological features of SARS-CoV-2-induced ARDS in humans, such as the disruption of lung tissues with apparent cell death, neutrophil infiltration, proteinaceous debris, hemorrhage, thrombi, and hyaline membranes-like changes, were rarely found in nasal inoculation of SARS-CoV-2 mouse model.^2,3^ Thus, these models failed to recapitulate the acute and severe form of lung injury, especially, its progression to ARDS. Therefore, the establishment of laboratory animal models for ARDS is important to support the studies for COVID-19 progression and treatment evaluation.

To establish the mouse model for SARS-CoV-2-induced ARDS, we infected the humanized hACE2-KI mice with C57BL/6 background by intratracheal instillation of SARS-CoV-2 (40 μl, 10^7^ PFU/ml), as demonstrated in Fig. 1a and in Supplementary Materials. The transgenic hACE2 mice with C57BL/6 background were provided by the National Institutes for Food and Drug Control in China.^2,4^ The studies were performed in the ABSL-4 facility of Kunming National High-level Biosafety Primate Research Center and were reviewed and approved by the Institutional Animal Care and Use Committee of Institute. The lung tissues from the infected mice were collected on 6 h, 1 day, 2 days, 3 days, and 5 days post inoculation of SARS-CoV-2 and were investigated for pathological changes. Grossly, lungs from the infected mice got the appearance of the bigger size, with the bilateral congestion and edema, and patches of dark-colored hemorrhage (Fig. 1b). Furthermore, the lung tissues showed an increase of basophilia and the reduction of airway spaces by the observation with hematoxylin and eosin (H&E) stain under light microscopy (Fig. 1c, d), which may result from the diffuse heavy inflammatory infiltrates in the lung tissues. Notably, this inflammation is often characterized by the disruption of lung tissues, with the disappearance of recognizable architecture (Fig. 1d). The dead cells were mostly seen in the background of the inflammatory cell infiltrates and were positively stained by using the terminal deoxynucleotidyl transferase dUTP nick end labeling (TUNEL) technique (Fig. 1e). The apparent cell death can be found as early as 6 h post inoculation of SARS-CoV-2.

**Fig. 1:**
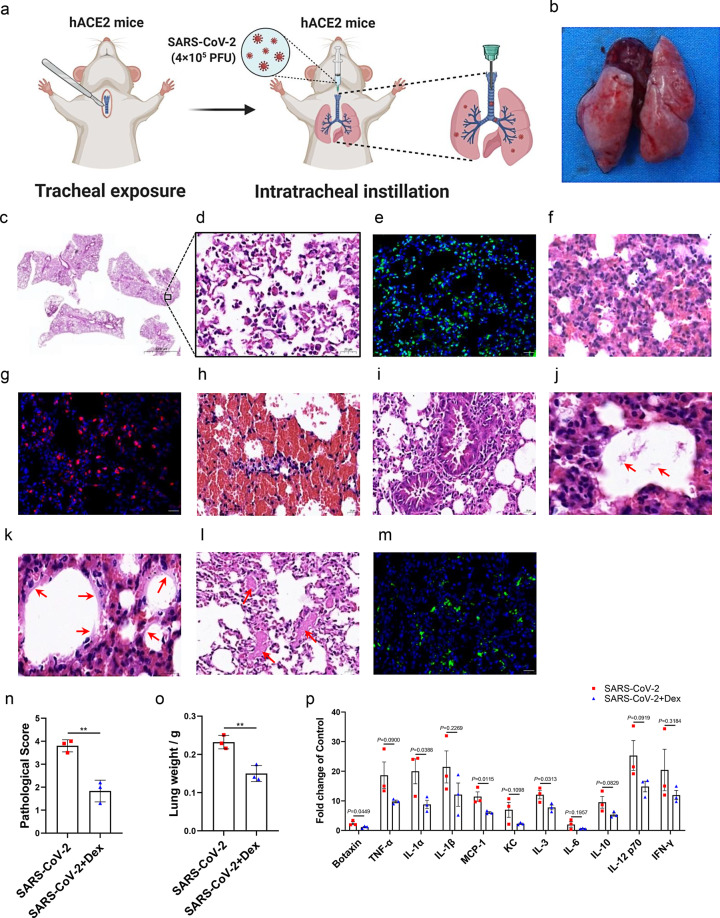
The characterization and treatment of a mouse model for SARS-CoV-2-induced acute respiratory distress syndrome (ARDS). **a** The establishment of a mouse model of ARDS. Illustration for routine tracheotomy and intratracheal instillation of SARS-CoV-2 in mouse (4 × 10^5^ PFU). **b** Morphology of the lung from the mouse 5 days after intratracheal instillation of SARS-CoV-2 (4 × 10^5^ PFU). **c**, **d** The pulmonary pathological changes in mice after intratracheal instillation of SARS-CoV-2 characterized by H&E staining. **e** TUNEL labeling was used to detect the cell apoptosis in lung tissue as described in Supplementary Materials. **f** The neutrophils were predominant cell infiltration with 60% of lung tissues consolidated as stained by H&E staining. **g** The neutrophils were stained positive for Ly6G. **h** Apparent hemorrhage, with the red blood cells filling the alveolar spaces in the section. **i** Inflammatory cells, red blood cells, and proteinaceous debris were found in the bronchioles. **j** Proteinaceous and cellular debris were found in the alveolar space (arrow). **k** Hyaline membranes-like changes lining alveolar cavities (arrow). **l** The thrombi were present in the vessels (arrow). **m** The immunostaining of SARS-CoV-2 spike protein in the lung section of a mouse with SARS-CoV-2-induced ARDS, as described in Supplementary Materials. **n** Pathological scores for mice from SARS-CoV-2-induced ARDS group or dexamethasone-treated group according to the scoring system (*n* = 3 mice). **o** The lung weights of mice from SARS-CoV-2-induced ARDS group or dexamethasone-treated group (*n* = 3). Mice were treated with dexamethasone as described in Supplementary Materials. **p** Cytokines changes in mice from SARS-CoV-2-induced ARDS group or dexamethasone-treated group (*n* = 3). Cytokines were measured as described in Supplementary Materials. Scale bars represent 2000 μm (**c**) or 20 μm (**d**–**m**). Statistical analysis of pathological score (**n**) and lung weight (**o**) was analyzed by Student’s unpaired *t* test, and cytokines changes (**p**) data were analyzed by one-way ANOVA. All error bars represent SEM about the mean. **P < 0.01

Inflammatory cell exudates were characterized by neutrophilic infiltration at the early stage. As shown in Fig. 1f, apparent neutrophils can be found in the alveolar or the interstitial space, with more than 60% of lung tissues consolidated at 6 h post inoculation of SARS-CoV-2 (Fig. 1f). Neutrophils were stained positive for Ly6G (Fig. 1g). Neutrophils remained predominant cell infiltration 24 h and 48 h post inoculation. The scattered monocytes/macrophages or lymphocytes gradually increased at 3 and 5 days post inoculation of the virus, but neutrophils were still frequently found. Red blood cells often exuded into the interstitial or the alveolar space (Fig. 1f). Even in some regions, there was apparent hemorrhage, with the red blood cells filling the alveolar spaces (Fig. 1h). The bronchioles often contained some inflammatory cells, red blood cells, and proteinaceous debris (Fig. 1i).

Except for the cellular exudates, the fluid exudates were characterized by tissue edema, or proteinaceous debris in the alveolar space (Fig. 1j), or the hyaline membranes-like changes (Fig. 1k). Although it has been reported that hyaline membranes are rarely found in the mice models, the hyaline membranes-like changes lining alveolar cavities were still found in this study (Fig. 1k). Sometimes, the thrombi were present in the vessels (Fig. 1l). In addition, the viral spike protein can be found in lung tissues (Fig. 1m). The pathologic changes such as the disruption of lung tissues, apparent cell death, thrombi, and hemorrhage were not found in LPS-induced or ventilator-induced ARDS in mice model. However, these pathologic changes were often consistent with those found in SARS-CoV-2 ARDS in humans.^4^ Therefore, our mice model may reflect the pathogenesis of SARS-CoV-2-induced ARDS in humans.

Based on the observation of the pathological changes in the lung tissues in the mice infected with the virus mentioned above and in the infected mice treated by drug (see below), we adopted a 5-grade scoring system to describe the severity of the lung damage from least severe to most severe: 0, 1, 2, 3, 4 as following: normal lung tissue was scored as 0; slight broadening of alveolar septa, with primarily neutrophilic infiltration were scored as 1; more broadening of alveolar septa, with neutrophil and monocyte/macrophage infiltration in the interstitial and/or the alveolar space, with less than 30% of lung consolidated, were scored as 2; apparent neutrophil and monocytes/macrophage infiltration in the alveolar or the interstitial space, with 30–60% of lung consolidated, were scored as 3. Even if 30–60% of lung consolidated were not reached, either one of the presence of the following pathological changes, such as the focal lung epithelial or endothelial cell death, or focal hemorrhage, or the proteinaceous debris in the alveolar space, or apparent edema in lung tissues, were still scored as 3. More than 60% of lung consolidated with neutrophil and monocyte/macrophage infiltration, was scored as 4. Even if there was less severe inflammatory cell infiltration in lung tissues, either one of the following pathological changes, such as the diffuse disruption of alveolar walls without the recognizable architecture, or the apparent lung epithelial or endothelial cell death, or apparent hemorrhage or thrombi in vessels, or the hyaline membranes-like changes, was also scored as 4. According to the scoring system, the mice treated with intratracheal instillation of SARS-CoV-2 reached an average score of 3.5–4 (Fig. 1n).

In the next of the experiment, we explored the potential application in the evaluation of the drugs for SARS-CoV-2 ARDS. Since dexamethasone has been recognized as an efficacy drug for SARS-CoV-2 ARDS in humans and reduced one of the third deaths,^1^ we treated the mice model with dexamethasone. We found that the mice treated with dexamethasone exhibited the alleviated pathological changes in the lung as scored from 1.3 to 2.2 (Fig. 1n). The average lung weight of mice treated with dexamethasone also decreased while compared with that of the SARS-CoV-2 group (Fig. 1o). ARDS in humans is reported to be accompanied by the occurrence of a “cytokine storm”. In the current SARS-CoV-2-induced ARDS mouse model, as shown in we also detected the increased levels of critical cytokines, such as TNF-α, IL-1α, IL-1β, MCP-1, etc. After the treatment of dexamethasone, most of the cytokines showed a tendency to decrease as shown in Fig. 1p. Among them, the level of IL-1α, MCP-1, and IL-3 were significantly decreased compared to that of the ARDS mouse model. Therefore, we suggested that this SARS-CoV-2-induced ARDS mouse model is sensitive to drug treatment and might be applicable to the drug screening and evaluation platform.

To summarize, we have successfully established an ARDS mouse model with intratracheal instillation of SARS-CoV-2, which exhibited the typical pathological changes in lung consistency with those found in SARS-CoV-2 ARDS in humans.^5^ Such model is sensitive to drug treatment of dexamethasone and might be useful in the future study of COVID-19 pathogenesis, immunity, and the evaluation of the drugs for SARS-CoV-2 ARDS.

## Supplementary information

Supplementary Materials for A mouse model for SARS-CoV-2-induced acute respiratory distress syndrome

## References

[1] Lin, P. et al. Coronavirus in human diseases: mechanisms and advances in clinical treatment. *MedComm*. 1–32 (2020). 10.1002/mco2.26 PMC7646666 33173860

[2] Bao L (2020). The pathogenicity of SARS-CoV-2 in hACE2 transgenic mice. Nature.

[3] Sun S (2020). A mouse model of SARS-CoV-2 infection and pathogenesis. Cell Host Microbe.

[4] Yang J (2020). A vaccine targeting the RBD of the S protein of SARS-CoV-2 induces protective immunity. Nature.

[5] Bian X-W, Team TC-P (2020). Autopsy of COVID-19 patients in China. Natl Sci. Rev..

