# Supplementary material for: A mouse model for SARS-CoV-2-induced acute respiratory distress syndrome
Source: Signal Transduct Target Ther. 2021 Jan 1;6:1. doi: 10.1038/s41392-020-00451-w (PMC7775436; doi:10.1038/s41392-020-00451-w)
Supplement: Supplementary file 1 — Supplementary Materials for A mouse model for SARS-CoV-2-induced acute respiratory distress syndrome [file 41392_2020_451_MOESM1_ESM.docx]

Supplementary Materials for

A mouse model for SARS-CoV-2-induced acute respiratory distress syndrome

Weiqi Hong^1+^, Jingyun Yang^1+^, Zhenfei Bi^1+^, Cai He^1^, Hong Lei^1^, Wenhai Yu^2^, Yun Yang^2^, Changfa Fan^3^, Shuaiyao Lu^2,4^, Xiaozhong Peng^2,4^, Xiawei Wei^1^

*1. Laboratory of Aging Research and Cancer Drug Target, State Key Laboratory of Biotherapy and Cancer Center, National Clinical Research Center for Geriatrics, West China Hospital, Sichuan University, No. 17, Block 3, Southern Renmin Road, Chengdu, Sichuan 610041, People’s Republic of China*

*2. National Kunming High-level Biosafety Primate Research Center, Institute of Medical Biology, Chinese Academy of Medical Sciences and Peking Union Medical College, Yunnan China*

*3. National Institutes for Food and Drug Control (NIFDC), Beijing, China*

*4.* *State Key Laboratory of Medical Molecular Biology, Department of Molecular Biology and Biochemistry, Institute of Basic Medical Sciences, Medical Primate Research Center, Neuroscience Center, Chinese Academy of Medical Sciences, School of Basic Medicine Peking Union Medical College, Beijing China*

^+^ These authors contributed equally to this work

* Correspondence: Shuaiyao Lu (lushuaiyao-km@163.com), Xiaozhong Peng (pengxiaozhong@pumc.edu.cn), Xiawei Wei (xiaweiwei@scu.edu.cn)

**This PDF file includes:**

Materials and Methods

**Materials and Methods**

**1. Virus and cells**

The SARS-CoV-2 isolate GD108# strain was used for mice challenge and was initially from Guangdong Provincial Center for Disease Control and Prevention. Stocks were amplified in Vero E6 cell line monolayers maintained in Dulbecco’s modified Eagle’s medium (DMEM, Thermo Fisher Scientific, USA), and infectious units were quantified by plaque assay.

**2. Mice and experiment protocol**

The transgenic hACE2 mice (8-10 weeks) with C57BL/6 background were provided by the National Institutes for Food and Drug Control ^1^. The intratracheal instillation was performed as described previously. Briefly, mice were anesthetized with 5% isoflurane, suspended by the front teeth and placed on a surgical board with a 45° angle. Open a small superficial incision in the midline of neck to expose the trachea, and 4×10^5^ PFU of SARS-CoV-2 in 40 μL of PBS was intratracheally instilled with a 29-gauge insulin syringe (Becton, Dickinson and Company, USA). After instillation, the overlying skin was closed with wound clips, and the animals were placed on a heating pad in their cage until they recovered from anesthesia. In dexamethasone-treated group, the mice were intraperitoneally (i.p.) injected with a total of 200 μL (5mg/kg) of dexamethasone immediately after the SARS-CoV-2 challenge, and were continuously injected once per day for five consecutive days. The mice were euthanized by isoflurane overdose on 6 hr, 1 day, 2 days, 3 days and 5 days post infection for serum collection and tissue processing. Lung tissues were collected and fixed in 10% phosphate buffered formalin for 5 days prior to removal from the BSL4 for processing. All procedures associated with animal study were reviewed and approved by the by the Institutional Animal Care and Use Committee of Institute of Medical Biology, Chinese Academy of Medical Sciences, and performed in the ABSL-4 facility of Kunming National High-level Biosafety Primate Research Center.

**3. Histological analysis and immunofluorescence**

Lungs were harvest and fixed in 10% phosphate buffered formalin for 5 days, embedded in paraffin and sectioned at 3 μm thickness. Sequential sections were stained with hematoxylin and eosin to assess pathology and lung damage.

Paraffin-embedded sections at 3 μm were used to immunofluorescence staining. Briefly, sections were baked, deparaffinized, rehydrated. Antigen retrieval was performed with an EDTA buffer at 95℃ for 15 min. After incubated with blocking buffer (5% normal goat serum) for 10 min at room temperature, the slides were incubated with primary antibodies overnight at 4℃. After five washes, the sections were incubated with secondary antibody at 37 ℃ for 1 hour. Primary antibodies used for immunofluorescence analysis included rabbit anti-myeloperoxidase (Abcam, ab9535), rat anti-Ly6G (Abcam, ab25377), rabbit anti-SARS-CoV-2 Spike (Sino Biological, 40589-T62), rabbit anti-Cleaved Caspase-3 (Cell Signaling Technology, #9661). Secondary antibodies included goat anti-rabbit IgG - H&L (Alexa Fluor® 488) (Abcam,ab), goat anti-rat IgG H&L preadsorbed (Alexa Fluor® 647) (Abcam, ab150167).

**4. TUNEL assay**

Paraffin-embedded sections at 3 μm were used to TUNEL assay. The TUNEL assay was performed according to the instruction of DeadEnd^TM^ Fluorometric TUNEL System (Promega, USA). The results were observed by fluorescence microscope (Leica, Germany).

**5. Chemokine and Cytokine analysis**

The serum from each mouse was collected at indicated points. Bio-Plex Pro Mouse Cytokine Grp I Panel 23-Plex (BIO-RAD) was used for analyzed cytokines in serum according to manufacturer’s protocol. Briefly, a total of 50 μL of 1:3 diluted serum samples were incubated with magnetic capture beads, washed, incubated with detection antibodies and SA-PE. The data were recorded on Luminex 200 and analyzed by xPONENT software.

**6.** **Statistical analysis.**

Statistical analyses were carried out using Prism software (GraphPad Prism 8.0). All data were analyzed using one-way ANOVA and Student’s unpaired t-test. Results are presented as the means ± SEM. P < 0.05 was considered significant (significance is denoted as follows: ns, no significance; *P ≤ 0.05; **P ≤ 0.01; ***P ≤ 0.001; ****P ≤ 0.0001).

**References**

1 Sun, S. H. et al. A Mouse Model of SARS-CoV-2 Infection and Pathogenesis. *Cell Host Microbe* **28**, 124-133.e124 (2020).
